# 3D shape analysis of the brain's third ventricle using a midplane encoded symmetric template model

**DOI:** 10.1016/j.cmpb.2016.02.014

**Published:** 2016-06

**Authors:** Jaeil Kim, Maria del C. Valdés Hernández, Natalie A. Royle, Susana Muñoz Maniega, Benjamin S. Aribisala, Alan J. Gow, Mark E. Bastin, Ian J. Deary, Joanna M. Wardlaw, Jinah Park

**Affiliations:** a School of Computing, Korea Advanced Institute of Science and Technology, Daejeon, South Korea; b Brain Research Imaging Centre, Department of Neuroimaging Sciences, University of Edinburgh, Edinburgh, UK; c Centre for Cognitive Ageing and Cognitive Epidemiology, University of Edinburgh, Edinburgh, UK; d SINAPSE (Scottish Imaging Network, A Platform for Scientific Excellence) Collaboration, Scotland, UK; e Department of Psychology, University of Edinburgh, Edinburgh, UK; f Psychology, School of Life Sciences, Heriot-Watt University, Edinburgh, UK; g Computer Science Department, Lagos State University, Nigeria

**Keywords:** Brain, Third ventricle, Shape analysis, 3D model, Aging, Atrophy

## Abstract

**Background:**

Structural changes of the brain's third ventricle have been acknowledged as an indicative measure of the brain atrophy progression in neurodegenerative and endocrinal diseases. To investigate the ventricular enlargement in relation to the atrophy of the surrounding structures, shape analysis is a promising approach. However, there are hurdles in modeling the third ventricle shape. First, it has topological variations across individuals due to the inter-thalamic adhesion. In addition, as an interhemispheric structure, it needs to be aligned to the midsagittal plane to assess its asymmetric and regional deformation.

**Method:**

To address these issues, we propose a model-based shape assessment. Our template model of the third ventricle consists of a midplane and a symmetric mesh of generic shape. By mapping the template's midplane to the individuals’ brain midsagittal plane, we align the symmetric mesh on the midline of the brain before quantifying the third ventricle shape. To build the vertex-wise correspondence between the individual third ventricle and the template mesh, we employ a minimal-distortion surface deformation framework. In addition, to account for topological variations, we implement geometric constraints guiding the template mesh to have zero width where the inter-thalamic adhesion passes through, preventing vertices crossing between left and right walls of the third ventricle. The individual shapes are compared using a vertex-wise deformity from the symmetric template.

**Results:**

Experiments on imaging and demographic data from a study of aging showed that our model was sensitive in assessing morphological differences between individuals in relation to brain volume (i.e. proxy for general brain atrophy), gender and the fluid intelligence at age 72. It also revealed that the proposed method can detect the regional and asymmetrical deformation unlike the conventional measures: volume (median 1.95 ml, IQR 0.96 ml) and width of the third ventricle. Similarity measures between binary masks and the shape model showed that the latter reconstructed shape details with high accuracy (Dice coefficient ≥0.9, mean distance 0.5 mm and Hausdorff distance 2.7 mm).

**Conclusions:**

We have demonstrated that our approach is suitable to morphometrical analyses of the third ventricle, providing high accuracy and inter-subject consistency in the shape quantification. This shape modeling method with geometric constraints based on anatomical landmarks could be extended to other brain structures which require a consistent measurement basis in the morphometry.

## Highlights

• Present a model-based approach to investigate the morphology of the third ventricle.
• Assess the regional deformations in relation to the atrophy of surrounding structures.
• Use a symmetric template model with the midplane definition for unbiased analysis.
• Achieve a robust surface modeling using a progressive surface deformation.
• Validate the method on a healthy aging sample with different clinical variables.

## 1 Introduction

The brain's third ventricle lies in the center of the brain and is surrounded by critical nuclei structures (i.e. hypothalamus and thalamus) that are often affected by dementia. Also surrounding them are important glandular structures (i.e. pituitary and pineal glands) that regulate homeostasis and affect seasonal functions. Therefore deformations or atrophy surrounding this cavity have been acknowledged as an indicative measure of atrophy progression in certain neurodegenerative (e.g. some subtypes of dementia and multiple sclerosis) and endocrinal diseases [1], [2], [3], [4], [5]. Recently, the third brain ventricle has been identified crucial in the early detection of Alzheimer's Disease [6], in the diagnosis of various movement disorders like progressive supranuclear palsy and Parkinson's Disease [7], and in the identification of various disorders in children like asymptomatic interhypothalamic adhesions [8] and craniopharyngiomas [9]. Moreover, knowledge of the brain third ventricle morphology is useful in investigating the presence of lesions around the intraventricular foramen, which are difficult to surgically remove for their important adjacent structures, helping to choose the appropriate surgical approach [10].

Due to its clinical importance, several methods have been proposed to assess the shape and volume of the third ventricle. To the best of our knowledge, clinical studies related to the enlargement of the third ventricle have specifically reported data based on either its volume or some direct measurement (e.g. diameter) obtained only from a particular MRI slice [2], [5], [11]. The volume of the third ventricle has been used in studies of brain aging [12], schizophrenia [13], [14], brain development [15], bipolar affective disorder [11], [16], and multiple sclerosis [3]. However, volumetric analysis has difficulty revealing the association between regional changes in ventricle structure and clinical factors. To investigate the ventricular enlargement at specific regions where the third ventricle meets the surrounding structures, a manual measurement of the width between the left and right lateral walls of the third ventricle has been proposed [2], [3], [5], [11], [17]. But manual measurements are susceptible to human errors. Also, variability in the assessment of the third ventricle makes reproducibility and protocol comparability difficult. For example, one study measured the third ventricle width on the coronal slice immediately posterior to the last slice where the anterior commissure was clearly visible [11]. In other studies, the third ventricle width has been measured by drawing a line perpendicular to the midline of this structure at an axial slice where it is most visible [2], [18]. Authors who have referred to the width of the brain third ventricle being assessed indistinctively on a coronal or axial slice, mention inter- and intra-operator variations and the different positions of the imaged head as causes for the low reproducibility of their results [18].

**Fig. 1:**
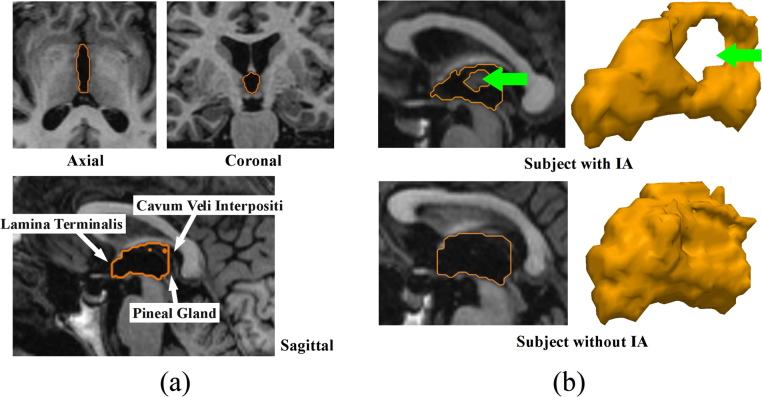
Third ventricle in structural magnetic resonance images. (a) small size and the low contrast of the surrounding structures (indicated by white arrows). Orange contours in the image are the third ventricle. (b) topological variations including the presence (above) and absence (below) of the inter-thalamic adhesion (IA). Green arrows indicate the holes of the third ventricle, where the IA passes through. (For interpretation of the references to color in this figure legend, the reader is referred to the web version of this article.)

Against the limitations of the third ventricle measurements just mentioned, the morphological analysis using parameterized shape models can be an alternative. There is increasing evidence that shape analysis provides useful information in the analysis of pathological and aging processes [19], [20], [21], [22], [23]. A study [24] presented a third ventricle shape registration method based on a heat-kernel representation. In shape analysis, the achievement of biologically meaningful shape representations, robustness to noise and small perturbations, and the ability to capture representative properties of the natural biological shape variation [25] are necessary challenges to overcome. In addition, shape modeling of the brain third ventricle is challenging due to the following reasons. First, the small size of the third ventricle and the low contrast of the membranes that separate this cavity from its surroundings yield rough surface boundaries in the segmentations and complicate the anatomical quantification of its morphology from the magnetic resonance (MR) images (Fig. 1(a)). Secondly, the topological inter-individual variations in the third ventricle, especially the presence/absence of the inter-thalamic adhesion (IA), which bridges the left and right thalami across the third ventricle, makes it difficult to assure shape correspondence between the third ventricle surfaces across individuals (Fig. 1(b)). Lastly, the third ventricle is surrounded by different brain structures, such as hypothalamus and thalamus, in the left and right hemispheres of the brain. Atrophy of the surrounding structures may change the shape and size of the third ventricle, and consistency in the anatomical landmarks is required to quantify its morphological changes in relation to the anatomical positions of the surrounding structures.

### 1.1 Our proposal

In this paper, we propose a novel approach to generate a 3D shape model of the brain third ventricle out from binary masks of this structure obtained from structural MR images. We endeavor that our model guarantees good inter-subject shape correspondence and provides robust geometric measures reflecting the wide range of morphological variations of the third ventricle. We newly introduce a template-based surface modeling approach using a symmetric template model with a midplane definition. For each individual, firstly the symmetric template model's midplane is optimally aligned to the brain's midsagittal plane (MSP) of the individual MR image. Then, the symmetric template is deformed non-rigidly to reconstruct the individuals’ third ventricle shape conserving point-wise correspondence between the template and the individual model throughout the whole surface. The topology problem, introduced by the inter-thalamic adhesion (IA), is tackled by geometric constraints that guide the deformable surface to have zero width at the sub-regions of the MSP where the IA passes through, as the left and right lateral walls contact each other at these sub-regions.

Through this model individualization process we can robustly obtain the smooth shape of the third ventricle after filtering out the rough boundaries and holes in the segmentation of the third ventricle, and compute the regional differences between the individual models. In addition, the symmetric template model and the model normalization based on the MSP allow the comparison of the magnitude and direction of the shape differences between left and right sides of the third ventricle.

We explore the usefulness of our model detecting morphological deformations in the brain third ventricle of community-dwelling older individuals, with the hypothesis that it is sensitive enough to show differences related to gender, fluid intelligence and general atrophy that are in agreement with clinical reports. We hypothesize that men will have wider and bigger third ventricles than women [12], [26] and that individuals with wider third ventricle will have smaller brains [27]. Previous studies that used third ventricle width as surrogate of thalamic atrophy [28], [29], found a significant association between this measurement and general cognition, independent of educational attainment [28]. Given that, as previously explained, measurements of third ventricle width vary, we expect variations in the association between the shape of the third ventricle and fluid intelligence (i.e. general cognitive ability) on the aging sample.

## 2 Method

**Fig. 2:**
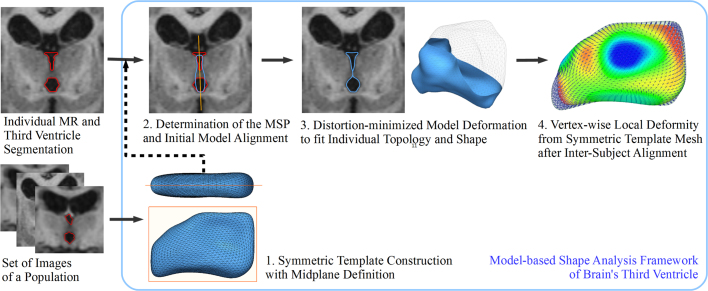
Representation of the proposed shape modeling and analysis framework for the brain's third ventricle. Abbreviations: MSP: midsagittal plane, MR: magnetic resonance imaging.

Given a set of binary masks of the third ventricle and T1-weighted brain MR images, we first build a symmetric template model representing a sample-wise mean shape of the third ventricle, then we project the template model onto individual binary masks via a MSP determination and a distortion-minimized model deformation to reconstruct the individual shapes of the third ventricle. Fig. 2 shows our shape modeling and analysis pipeline. In the following section, we describe each step of the pipeline.

### 2.1 Symmetric template model of the third ventricle with midplane definition

**Fig. 3:**
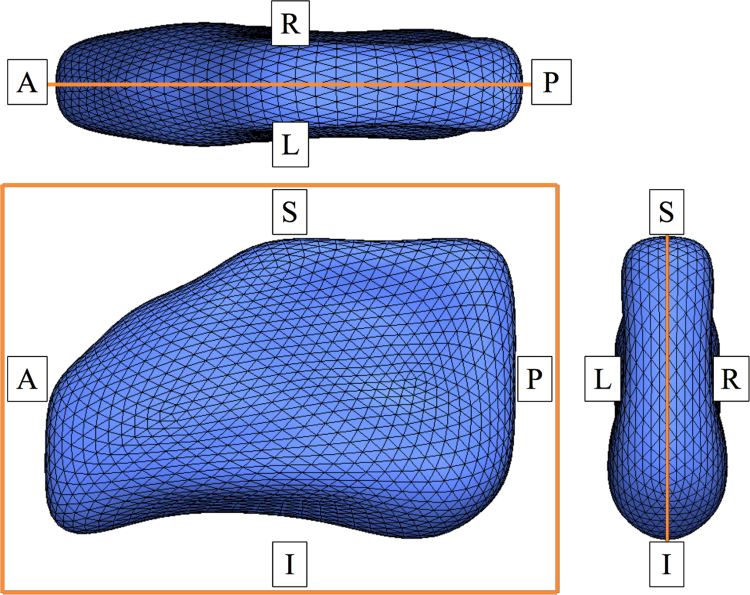
Symmetric template model of the third ventricle including the symmetric mesh (blue wireframed surface) and its mid-plane (orange solid outline). (For interpretation of the references to color in this figure legend, the reader is referred to the web version of this article.)

Our brain third ventricle template model consists of a symmetric triangular mesh and its midplane. The symmetric mesh is used as a shape prior in the individual modeling process for quantifying inter-subject shape differences. To represent the generic shape characteristics of the brain's third ventricle, the mesh is constructed out of the binary masks from the study population. To generate the template mesh we, firstly, find the individual MSPs. To determine the position and orientation of the MSP in native image space, we rigidly register individual T1-weighted images to a standard brain atlas on the Talairach space. The MSP in the Talairach space is defined with the direction vector of the *x*-axis (1, 0, 0) as the plane normal and the global center (0, 0, 0) as plane center [30]. We use the ICBM-152 atlas [31] and FLIRT (FMRIB's Linear Image Registration Tool, Version 5.0) [32], [33] for this registration process. The MSPs in the registered images need to be checked and manually corrected. Then, we determine the inverse transformation of the rigid registration to the MSP in standard space to estimate each MSP in the native space. After finding the MSP for each image, we obtain a symmetric shape of the third ventricle from the collected images via non-rigid registration between the input binary masks and their left-right-flipped images with the individual MSPs. This symmetric shape construction follows the unbiased symmetric template construction process proposed in [31]. From the symmetric shape, we build a triangular mesh using marching cubes and a uniform resampling algorithm from two open source software applications (Medical Image Interaction Toolkit (http://www.mitk.org, Ver. 2013.12) and OpenFlipper (http://www.openflipper.org, Ver. 1.3)). Fig. 3 shows the symmetric third ventricle mesh with its midplane of an aging population, described in Section 3.

### 2.2 Individual shape modeling of the third ventricle

#### 2.2.1 Model initialization with MSP matching

To model the third ventricle shape of each individual we, firstly, optimally and rigidly align the symmetric template mesh to the target structures (i.e. native image space). Once each individual MSP is identified as described in the previous section, the third ventricle mesh, in standard space, is optimally aligned to the individual target structure via a two-step rigid registration. In the first step, we compute a rigid transformation matching the center and plane normal vector of individuals’ MSP and the template midplane. In the second step, the template mesh is transformed to the optimal position and orientation using the iterative closest point algorithm [34], which computes a rigid transformation minimizing the geometric distances between the template mesh and the target shape. In this second step, we used the voxel mesh extracted from each individual binary mask as the point cloud. Although the iterative closest point algorithm computes the rigid transformation with 6 degrees of freedom, we limit the rigid registration to 3 degrees of freedom: a rotation about the normal vector of the MSP and a translation about the *y*- and *z*-axes of the MSP. This guarantees that the center of mass of the third ventricle model stays on the MSP and that the normal vector of the midplane in the template model is consistent with that of the MSP.

#### 2.2.2 Non-rigid shape reconstruction via a progressive surface deformation

Once the template shape model is aligned to the native image space, the individual shape of the third ventricle is obtained through a non-rigid deformation of the aligned template mesh using the “Progressive Surface Deformation” method [23]. Through this method, each vertex of the template mesh is propagated into the closest image boundaries reconstructing the target shapes from the mesh structure of the template model. The method minimizes the distortion of the point distribution of the template mesh, produced by arbitrary size and shape variations of the targets. This minimization is achieved through a smooth propagation of external forces across the entire surface. This is achieved by a large-to-small scale deformation based on an extended Laplacian operator with N-ring neighborhood and a flexible weighting scheme of surface model rigidity [23]. The surface deformation method can establish a consistent mapping between template mesh and target shapes in the binary volumes, and the individuals’ shapes can be compared via a transitive relation with the template mesh. The robustness and accuracy of the method in generating smooth shape models with point correspondence has been validated in the hippocampi [23]. However, unlike the hippocampi, the third ventricle has a particular topological variation between individuals characterized by the presence/absence of IA, and the morphological analysis of the third ventricle requires an explicit separation of left and right regions of the third ventricle given by the brain asymmetry.

Therefore, for the third ventricle shape modeling, we additionally implement midplane-based constraints to the Progressive Surface Laplacian-based Deformation framework. Briefly, as explained in details in [23], this framework's energy function is defined as per Eq. (1):

(1) $$E(V^{\prime })=\sum_{i=1}^{n}∥\alpha_{i}(L(v_{i}^{\prime })-L(v_{i})){∥}^{2}+\sum_{i=1}^{n}∥b_{i}-v_{i}{∥}^{2}$$

In Eq. (1), *V*′ is the set of new vertex coordinates $v_{i}^{\prime }$, *n* is the number of vertices and *L* is a discrete operator of the Laplacian coordinates $L(v_{i})$. The Laplacian coordinates represents local geometry of the surface at each vertex as the deviation of the vertices from the centroid of their neighbors. $v_{i}$ are the coordinates of each vertex and *α*_*i*_ is the model rigidity parameter for them. *α*_*i*_ is determined dynamically with respect to the magnitude of the external force ($b_{i}-v_{i}$), which is propagated linearly to the neighbors with respect to *α*_*i*_. *b*_*i*_ is a geometric constraint that determines the desired positions of each vertex during the surface deformation process (mathematically defined in [23], [35]). The surface deformation is achieved by iteratively finding the optimal vertex positions (*V*′) minimizing the energy function (Eq. (1)) as is explained in details in [23], [35], [36]. This deformation gradually reduces the shape difference between the third ventricle mesh and the target volumes while preventing the distortion of the differential coordinates.

In the iterative surface deformation, each vertex of the third ventricle mesh is individually transformed to the desired vertex positions, denoted as *b*_*i*_ in Eq. (1), which are mainly the positions of the closest image boundary in the binary masks on the direction of the surface normal or on the opposite direction. However, when IA is observed, *b*_*i*_ is complemented by the midplane-based constraints to explicitly recover the region where the IA exists.

Here, we apply two types of the midplane-based constraints to restore the IA region during the model deformation. The first is a passive constraint that uses the midplane, which is mapped to the brain's MSP, to prevent the vertices from penetrating it. The second is an active constraint to attract the vertices at the IA region to the midplane during the model deformation. When there is an empty space between vertex $v_{i}$ at the lateral walls of the third ventricle mesh and the midplane, $v_{i}$ is attracted to its projected position on the midplane. This active constraint is applied to the model, when the distance between $v_{i}$ and the midplane is less than a threshold. The latter is determined as twice the diagonal length of an image voxel. Consequently, it drives the complete contact of the left and right side surfaces of the third ventricle mesh at the sub-regions of the midplane where the IA passes through. Vertex crossing between them is also prevented during the surface deformation. Our shape modeling process of the individual third ventricle is summarized in Algorithm 1.

Algorithm 1 Individual shape modeling for brain's third ventricle.

**Input:** MR image (*I*) and brain third ventricle binary mask (*TVBM*).

**Output:** Deformed template mesh (*S*′).

(a) **Symmetric template initialization**:
  1. Determine the brain midsagittal plane (*M*′) of *I* by registering *I* to a standard brain atlas;
  2. Move a symmetric template mesh (*S*) using a transformation matching template's midplane (*M*) to *M*′;
  3. Optimally align *S* to *TVBM* using a rigid transformation (translation and rotation along plane normal of *M*′) minimizing the surface distance between *S* and *TVBM*.
(b) **Non-rigid deformation of** *S*:
  In each iteration of the progressive surface deformation [23]:
  1. Update vertex normals for each vertex $v_{i}$ of *S*;
  2. Compute external force attracting $v_{i}$ to the closest boundary of target ventricle in *TVBM*;
  3. Check empty space between $v_{i}$ at the lateral wall of *S* and *M*′;
  4. If empty, update the external force for $v_{i}$ using a vector from $v_{i}$ to its closest point on *M*′ (active constraint);
  5. Update the vertex positions of *S* by solving a linear system of Laplacian deformation framework [23];
  6. Fix the position of $v_{i}$ at the lateral wall of *S* penetrating *M*′ with the closest point of $v_{i}$ to *M*′ (passive constraint).

#### 2.2.3 Model-based shape measures for the third ventricle

To quantify the structural differences between the individuals’ third ventricles, we measure the non-rigid shape difference of the third ventricles using the vertex transformation from the symmetric template model. In this measurement, we first normalize the individualized models to the symmetric template model via a rigid Procrustes alignment followed by an isotropic rescaling by a scaling factor (e.g. head size). In this process, the rigid Procrustes alignment is restricted to 3 degrees of freedom transformation including a rotation about the normal vector (*x*-axis) of the midplane and a translation about the *y*- and *z*-axes of the midplane like the model initialization described in Section 2.2.1. The vertex-wise deformity is the signed Euclidean norm of the vector representing the displacement between an individual mesh and the template mesh, projected on the vertex normal of the symmetric template mesh, which expresses the direction of local shape changes. Owing to this normalization process, the vertex-wise deformity can measure the asymmetric shape between the left and right halves of the third ventricle as well as the regional shape differences of the third ventricle between individuals.

The width and asymmetry of the third ventricle are determined from width maps that encode the distance between left and right surfaces of the third ventricle and the MSP. For this, the MSP is regularly sampled by a set of points on intervals of 0.5 mm on the *x* and *y* axes in the Talairach space. The width at a sampled point is the Euclidean distance between the intersection points of rays normal to the MSP and the third ventricle surface. Being *LW*(.) and *RW*(.) the width maps of left and right sides respectively and S the set of all sample points, the third ventricle width *TVW*_*i*_ and asymmetry *TVA*_*i*_ at each sample point *p*_*i*_ are calculated as:

(2) $${\mathrm{TVW}}_{i}=\mathrm{LW}(p_{i})+\mathrm{RW}(p_{i})$$

(3) $${\mathrm{TVA}}_{i}=\frac{\left|\mathrm{LW}(p_{i})-\mathrm{RW}(p_{i})\right|}{\max_{p_{i}\in S}({\mathrm{TVW}}_{i})}$$

#### 2.2.4 Validation of the proposed third ventricle modeling method

We validated our method using synthetic data and a sample from a study of cognitive aging. We constructed synthetic data introducing various degrees of asymmetry, surface roughness and sizes of IA. These analyses appear in the Supplementary material. On the aging sample we performed the following experiments: (1) validation of accuracy in reconstructing the individual shape of the individuals’ brain third ventricle using shape similarity metrics and (2) evaluation of the feasibility of the proposed method in detecting morphological variations of the third ventricle related to gender, general brain atrophy and fluid intelligence, comparing our results with existing clinical and cognitive reports [12], [26], [37], [27]. These analyses appear in the following section.

## 3 Evaluation of our model on a sample from a study of cognitive aging

### 3.1 Materials and methods

#### 3.1.1 Sample

We used magnetic resonance imaging (MRI) data from 51 participants (33 women and 18 men) randomly selected from The Lothian Birth Cohort 1936 Study [38] (www.lothianbirthcohort.ed.ac.uk). The data used in our experiments were obtained at mean age of 72.7 years (standard deviation = 0.7 years). Written informed consent was obtained from all participants under protocols approved by the Lothian (REC 07/MRE00/58) and Scottish Multicentre (MREC/01/0/56) Research Ethics Committees.

#### 3.1.2 MRI scans

Individuals’ brains were imaged in a GE Signa HDxt 1.5T clinical scanner (General Electric, Milwaukee, WI, USA) with a manufacturer supplied 8-channel phased-array head coil following a scanning protocol described in detail elsewhere [39]. This work uses measurements obtained from the T1-weighted, T2*-weighted and fluid attenuation inversion recovery (FLAIR) structural MRI sequences. The T1-weighted sequence was used to generate the binary masks of the third ventricle and the MSP. It was acquired in coronal orientation, had inversion, echo and repetition times of 500/4/9.8 ms respectively, flip angle of 8 degrees, slice thickness of 1.3 mm, bandwidth of 122 Hz/pixel and voxel dimension of 1.0 mm × 1.0 mm × 1.3 mm. The T2*-weighted sequence was used to generate the intracranial and brain tissue volumes. It was acquired in axial orientation, had echo and repetition times of 15 and 940 ms respectively, slice thickness of 2 mm and voxel dimension of 1.0 mm × 1.0 mm × 2.0 mm. The FLAIR sequence, combined with the T2*-weighted sequence, was used to generate brain tissue volume. FLAIR acquisition parameters were: inversion, echo and repetition times: 2200, 140 and 9000 ms respectively, slice thickness 4 mm and voxel size of 1.0 mm × 1.0 mm × 4.0 mm.

#### 3.1.3 Third ventricle measurements

We generated the binary masks of the third ventricle, following the segmentation procedure described previously in [27]. The results were visually assessed by a trained image analyst and manually rectified in case of inaccuracies. We generated the symmetric template model of the third ventricle out from the binary masks following the process described in Section 2.2.1. The midsagittal plane was obtained from the T1-weighted images using the linear registration tool of the FMRIB software library as described previously (Section 2.2.1), and also corrected manually afterwards in cases where this was required.

#### 3.1.4 Head and brain tissue volume measurements

We used intracranial volume (i.e. contents within the inner skull table including brain tissue, cerebrospinal fluid, veins and dura) as a measure of the individual's head size unaffected by age-related changes. It was extracted semi-automatically using the T2*-weighted sequence, with the Object Extraction Tool in Analyze™10.0 followed by manual editing [40]. Intracranial volume was used to correct the size of the individualized third ventricle models prior to obtaining the model-based shape measurements. We obtained brain tissue volume (BTV) following the procedure described in [27]. BTV, adjusted by head size (i.e. intracranial volume), was used as marker for general brain atrophy in the analysis. Age in days at the time of the MRI scan and gender were also used in the statistical analyses.

#### 3.1.5 Assessment of the fluid intelligence at age 72

All study participants underwent a wide range of cognitive tests as described in [38]. We used six subtests of the Wechsler Adult Intelligence Scale (WAIS-III^*UK*^) [41]: Digit Symbol, Digit Span Backward, Symbol Search, Letter-Number Sequencing, Block Design and Matrix Reasoning, to generate a measurement of fluid intelligence through Principal Component Analysis as described in [42]. The variable generated (*fi*) was continuous and normally distributed throughout the sample.

### 3.2 Experiments

#### 3.2.1 Accuracy evaluation of the individual shape modeling method

We computed the shape similarity between individualized models and binary masks for the 51 datasets of the sample using five metrics: (1) the volumetric similarity index (i.e. Dice coefficient, Dc) [43], (2) the symmetric mean distance (Md), (3) the symmetric Hausdorff distance (Hd), (4) the relative volume difference and (5) the volume overlap error [44]. To compute the Dc between the individualized meshes and the segmentations, we converted the meshes into binary images with the same voxel size as the original binary masks (i.e. the ones used to generate the models). We computed the Md and Hd between the voxel meshes, generated from the binary masks, and the individualized models, and the relative volume difference and volume overlap error between the original binary masks and the binary masks obtained from converting the meshes to volumes. We also analyze the correlation and perform the Bland–Altman analysis [45] between these volumetric measurements.

#### 3.2.2 Feasibility assessment of morphological variability in the sample

**Fig. 4:**
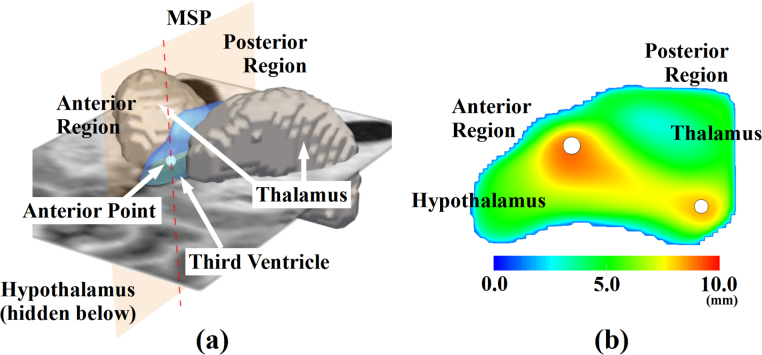
Third ventricle width measurement. (a) 3D illustration including the third ventricle mesh, midsagittal plane (MSP) and the anterior point (white dot) of the maximum width at the anterior region. (b) distance map between left and right walls of the third ventricle on the MSP. White dots indicate the points of TVW measurement at the anterior and posterior regions. (For interpretation of the references to color in this figure legend, the reader is referred to the web version of this article.)

We performed statistical analyses using local shape deformity, brain third ventricle volume (TVV), width (TVW), asymmetry (TVA), brain tissue volume (BTV), *fi* and gender. The TVV was computed by summing the volume of voxels of the third ventricle in the binary masks. The TVW was measured at the anterior and posterior regions of the third ventricle where it contacts with two different structures: thalamus and hypothalamus respectively as Eq. (2) and Fig. 4 show. For reporting, the anterior and posterior TVW quantify the maximum distances between lateral left and right walls of the third ventricle. The anterior TVW corresponds to the TVW in [46]. We also quantified the average width of the third ventricle with respect to the MSP.

We used linear regression to examine: (1) whether gender influences the shape and size measurements of the third ventricle, (2) whether general brain atrophy (i.e. reduction in brain size) is associated with the shape and size measurements of the third ventricle, and (3) whether fluid intelligence at age 72.7 years is related or not to brain third ventricle morphology and size. We anticipated that: (1) gender is associated with third ventricle measurements with men having larger and wider third ventricles than women [12], [26], (2) third ventricle measurements are positively associated with total brain atrophy: the bigger the third ventricle, the smaller the brain tissue volume and, therefore, the higher the total brain atrophy [27], and (3) some regions of the third ventricle may be associated with cognition as the latter relates with the morphology of the surrounding structures [47], [28]. To test our hypotheses we implemented robust univariate linear regression using the function “robustfit” from MATLAB R2013b Statistical Toolbox. The first regression equation was run 3 times (i.e. one for each third ventricle measure: width, volume and deformation) and included each third ventricle measure as dependent variable and gender as the independent variable, with age as covariate. The second regression equation was also run 3 times and it had BTV as independent variable and age and gender as covariates. The third regression equation was also run 3 times and it had *fi* as dependent variable, the third ventricle measurement as independent variable and age and gender as covariates.

### 3.3 Experiment results

#### 3.3.1 Accuracy of the reconstructed shape models

**Fig. 5:**
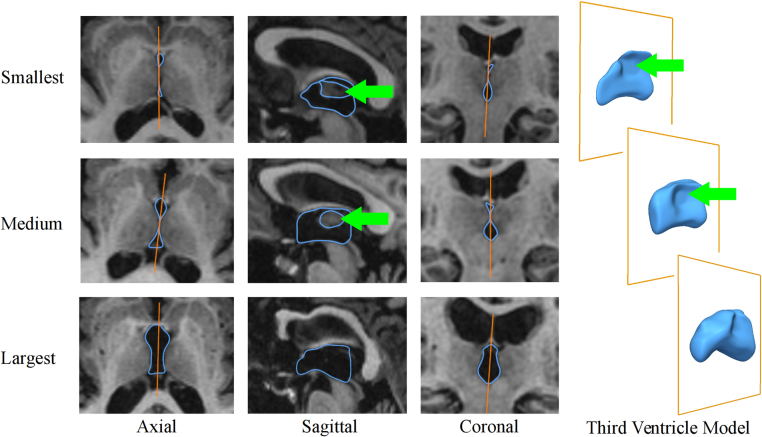
Individualized third ventricle models of individuals with the smallest, medium and largest third ventricle. Orange solid lines indicate the midsagittal plane and blue surfaces and contours indicate the third ventricle mesh. Green arrows indicate the contact of the lateral surfaces of the third ventricle mesh at the inter-thalamic adhesion. (For interpretation of the references to color in this figure legend, the reader is referred to the web version of this article.)

**Fig. 6:**
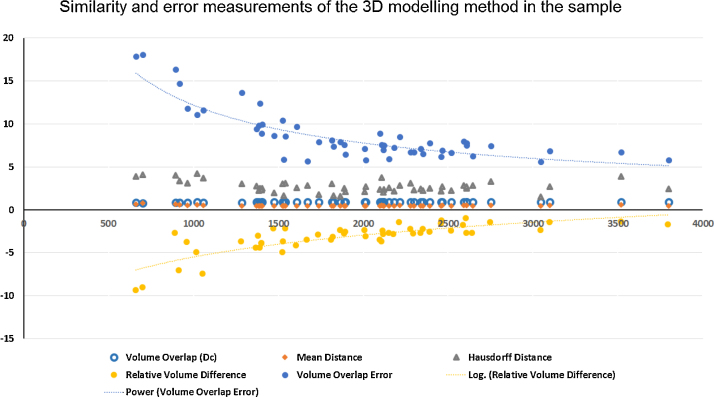
Similarity and error measurements of the brain third ventricle 3D modeling method. The volumes of the original binary masks in mm^3^ are represented in the horizontal axis. The vertical axis corresponds to the values of each similarity measurement given on each of their respective units. The Dice coefficient, mean distance and Hausdorff distance have a linear behavior across the sample. The relative volume difference and the volume overlap error decrease with the increase of the volume of the third ventricle following power and logarithmic distributions respectively, which are also represented as dashed lines.

Fig. 5 shows the individualized third ventricle models, generated from the binary masks of the individuals having the smallest, medium and largest third ventricles. Table 1 shows the average and standard deviation of Dc, Md and Hd for all individuals and for the individuals grouped according to the presence/absence of IA. The average Dc was 0.913, the Md was 0.485 mm, and the Hd was 2.682 mm. From the datasets having IA (21/51 individuals), the metrics generated lower values of Dc (Dc: 0.890, Md: 0.504 mm and Hd: 2.943 mm) than the values from the individuals not having IA (Dc: 0.929, Md: 0.472 mm and Hd: 2.499 mm). The behavior of these three metrics across the sample was linear and no bias towards the third ventricle size was observed as Fig. 6 shows.

The relative volume difference and the volume overlap error, both calculated considering the original binary masks and the binary masks obtained from converting the meshes to volumes as previously explained, decreased exponentially with the increase of third ventricle volume across the sample (Fig. 6). The median and interquartile range of these measurements were −2.806 mm^3^ (1.421 mm^3^) and 7.588 (2.819) respectively.

**Fig. 7:**
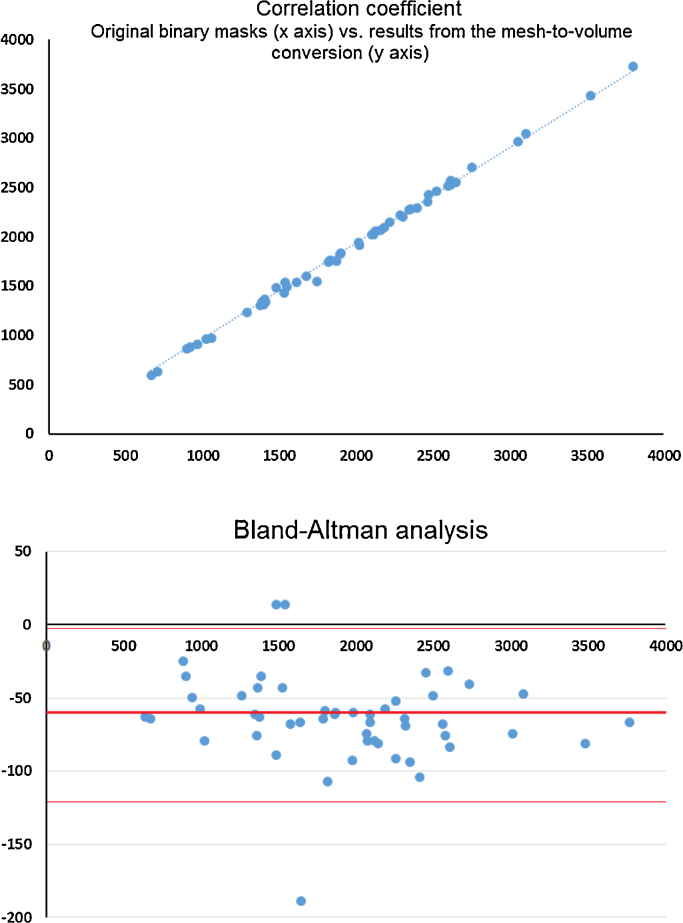
Correlation between the volumes of the original binary mask (horizontal axis) and the volumes of the binary masks resultant from converting the meshes to binary volumes (vertical axis) (top). Bland–Altman plot comparing both volumes (below).

To further explore the implications of the volume overlap error and the relative volume difference we calculated the correlation between the volume obtained from the individual third ventricle meshes and the binary masks (Fig. 7) The correlation coefficient was 0.970 (*R*-squared value 0.998). The Bland–Altman analysis did not show bias towards the third ventricle size, but that, comprehensible, the original binary masks were of slightly larger volumes than the masks obtained from converting the meshes to volumes. Considering the voxel size (1.0 mm × 1.0 mm × 1.3 mm) and the fact that the binary masks from this sample have rougher boundaries, the metrics indicate that our method restored individual shapes of the third ventricle from the binary masks accurately.

#### 3.3.2 Statistical analysis using 3D shape models, ventricular volume and width

**Fig. 8:**
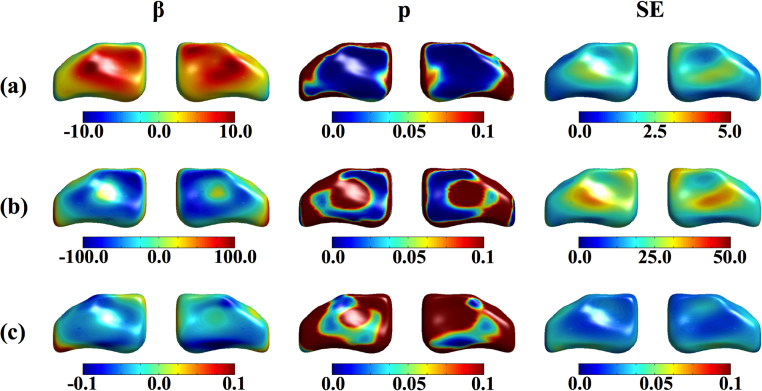
Results from the regression analysis between the vertex-wise deformity and the clinical variables mapped on the symmetric template mesh of the brain third ventricle. (a): regression with gender, (b): regression with brain tissue volume, and (c): regression with fluid intelligence at age 72.7 years. SE means the standard error of the regression. Left and right hand side views are shown. (For interpretation of the color in the figure, the reader is referred to the web version of this article.)

Table 2 presents the results of the regression analyses involving TVV and TVW. Fig. 8 presents the results of the regression analyses involving the vertex-wise shape deformity, reflecting asymmetry patterns. In general TVA was not associated with any of the clinical variables analyzed (i.e. gender (*p* = 0.209), BTV (*p* = 0.539) and *fi* (*p* = 0.455)). In the first model, as anticipated, the volume and width of the third ventricle were significantly associated with gender. Men had relatively larger third ventricles than women. These differences were significant throughout the lateral walls (Fig. 8(a)) in agreement with previous reports [12], [26]. In the second model, TVW were significantly and negatively associated with BTV: i.e. the TVW on the anterior region increased as the BTV decreased (more brain atrophy). However, the TVV was not significantly associated with the BTV (*p* > 0.05). In relation to the vertex-wise deformity, we found that ventricular contraction (*β* < 0 and *p* < 0.05 on major regions of the left and right surfaces) was related to BTV increase, while ventricular enlargement was observed (*β* > 0 on the front and rear surfaces) only in reduced areas of the anterior and posterior regions of the third ventricle (Fig. 8(b)) in relation with the same global brain atrophy indicator, but this latter association did not survive multiple comparisons correction. An aging study found that the width of the posterior regions of the third ventricle was significantly related (*p* < 0.05) to the overall brain atrophy [37] unlike its anterior regions (*p* > 0.05). The third model yielded similar, although slightly more modest, results as the second model in relation to the association between the clinical parameter (*fi* in this case) and TVW and TVV. In relation to the vertex-wise deformity, we found that ventricular contraction (*β* < 0 and *p* < 0.05 on some regions of the superior left and inferior right surfaces) was related to better general cognition.

## 4 Discussion

In this paper, we propose a model-based approach using the symmetric template surface model and its midplane for the morphology analysis of the third ventricle. We model and align the ventricular surfaces using a symmetric template model with respect to the MSP of each individual brain to investigate the morphological changes of the third ventricle in relation to brain atrophy in the left and right hemispheres separately. The progressive model deformation used helps to build a pairwise dense correspondence between the template mesh and the target structure against large size and shape variations in the third ventricle. The topological variation, reflected in the presence/absence of the IA, is tackled by dividing the third ventricle by its midplane and explicitly restoring the region where the IA passes through at the midplane using the midplane-based deformation constraint. We complement our proposal with the introduction of a shape analysis that uses the vertex-wise deformity and the midplane-based measures (i.e. third ventricle width and asymmetry).

Our surface modeling method allows accurate adjustments of the third ventricle models on an older sample with an accuracy of 91% volume overlap. We demonstrated the feasibility of our framework in the detection of morphological differences in this brain structure with respect to gender and two brain health indicators (i.e. brain tissue volume and fluid intelligence). Our analyses revealed the validity of our method as it generated results consistent with previous reports. However, due to the sample size (51 individuals), the patterns of the morphological differences of the third ventricle shown cannot be generalized. They need further confirmation on a larger sample and testing on large groups including healthy and cognitively impaired individuals. Nevertheless, our results show that this model-based approach helps to understand the complex patterns of the morphological changes in the third ventricle and its specific sub-regions in relation to gender, the atrophy of the surrounding structures and cognition.

Third ventricle width has been used previously as surrogate of thalamic volume [28], [29]. A study that assessed the predictive value of different conventional MRI-parameters both for overall and domain-specific cognitive performance concluded that thalamic volume has greater impact on memory function [48]. However, another study [47] found that thalamic volume was, instead, predictive of processing speed. Due to the small sample size used to evaluate the applicability of our modeling approach, we did not explore the association of third ventricle shape morphology and specific cognitive domains. It is possible that the difference between these results, both obtained on samples of patients with multiple sclerosis, were consequence of the difference between the measures used to represent the thalamic volume. The use of a consistent measure of third ventricle width, such as the one that our modeling framework provides, will help in clarifying apparent contradictions like the one mentioned above.

Our method provides solid geometric and anatomical criteria of the shape quantification of the third ventricle, thus guaranteeing reproducibility and consistency in the shape analysis between individuals, unlike previous approaches based on manual measurements. However, currently our method is not fully automated due to the need of confirmation and correction of the MSP by experts. In this study, we performed manual correction to secure the accurate position and orientation of the MSP for each subject. In [49], authors compared various methods for extracting the MSP, classified into feature-based, global symmetry based and local symmetry based methods. The feature-based methods for MSP extraction may be more suitable for our purpose, as they are based on detecting the known anatomical features of the MSP, such as the interhemispheric fissure, without the assumption of bilateral symmetry on the brain. However, exploring ways of automating the extraction of the MSP needs further research, which is out of the scope of this study.

The strengths of this study are: the surface modeling and shape assessment that allowed to accurately reconstruct the individual shape details across the large variability in the shape and topology of the third ventricle and quantify the complex patterns of the morphological differences between individuals with geometric and anatomical definitions. In addition, the statistical analyses performed on data from an aging sample that allowed us to test the feasibility of our shape assessment framework for studying associations between the morphology of the third ventricle and overall brain atrophy and cognition, confirming results obtained from previous studies that used conventional measurements of this brain structure or indirect measurements.

## Conflict of interest

No authors have been paid for this work, and all declare there are no conflicts of interest.

## Figures and Tables

**Table 1:** Shape similarity between the individualized models and the binary masks of the manual segmentation from a study of aging.

	*N*	Dice coefficient (Dc)	Mean distance (Md)	Hausdorff distance (Hd)
Individuals without IA	30	0.929 ± 0.010	0.472 ± 0.033	2.499 ± 0.563
Individuals with IA	21	0.890 ± 0.035	0.504 ± 0.096	2.943 ± 0.716
All individuals	51	0.913 ± 0.030	0.485 ± 0.067	2.682 ± 0.662

IA.: inter-thalamic adhesion, data for each element: mean ± SD.

Unit of mean and Hausdorff distance: mm.

**Table 2:** Regression analysis with the model-based measures and the clinical variables.

	TVV	Average TVW	Anterior TVW	Posterior TVW
Gender	*β* = 667.782	1.481	0.918	1.586
*p* = 0.002*	0.002*	0.012*	0.001*
SE = 205.262	0.439	0.353	0.456

BTV	*β* = −4119.835	−12.938	−7.136	−14.249
*p* = 0.135	0.022*	0.137	0.014*
SE = 2709,063	5.479	4.716	5.624

*fi*	*β* = −0.0004	−0.221	−0.226	−0.202
*p* = 0.039*	0.045*	0.091	0.057
SE = 0.0002	0.107	0.131	0.103

TVV: third ventricle volume (mm^3^), TVW: third ventricle width (mm), *fi*: fluid intelligence at age 72, and BTV: brain tissue volume corrected by head size.

TVV and BTV were corrected for the head size.

Data for each element: beta (*β*, non-standardized coefficient), *p*-value and standard error (SE).

* *p* < 0.05.
