# Supplementary material for: 3D shape analysis of the brain's third ventricle using a midplane encoded symmetric template model
Source: Comput Methods Programs Biomed. 2016 Jun;129:51–62. doi: 10.1016/j.cmpb.2016.02.014 (PMC4841787; doi:10.1016/j.cmpb.2016.02.014)
Supplement: Supplementary file 1 [file mmc1.pdf]

3D Shape Analysis of the Brain's Third Ventricle using a Midplane encoded Symmetric Template Model: Supplementary Material

Jaeil Kim[a], Maria del C. Valdes Hernandez[b,c,d], Natalie A. Royle[b,c,d], Susana Muñoz Maniega[b,c,d], Benjamin S. Aribisala[b,c,d,g], Alan J. Gow[c,f], Mark E. Bastin[b,c,d], Ian J. Deary[c,d,e], Joanna M. Wardlaw[b,c,d], Jinah Park[a,*]

[a] *School of Computing,Korea Advanced Institute of Science and Technology, Daejeon, South Korea*
[b] *Brain Research Imaging Centre, Department of Neuroimaging Sciences, University of Edinburgh, Edinburgh, UK*
[c] *Centre for Cognitive Ageing and Cognitive Epidemiology, University of Edinburgh, Edinburgh, UK*
[d] *SINAPSE (Scottish Imaging Network, A Platform for Scientific Excellence) collaboration, Scotland, UK*
[e] *Department of Psychology, University of Edinburgh, Edinburgh, UK*
[f] *Psychology, School of Life Sciences, Heriot-Watt University, Edinburgh, UK*
[g] *Computer Science Department, Lagos State University, Nigeria*

This supplementary material accompanies the paper "3D Shape Analysis of the Brain's Third Ventricle using a Midplane encoded Symmetric Template Model". It provides experimental results to show the robustness of the proposed method against A) asymmetric deformations and B) topological variations in the third ventricle.

Evaluation of Robustness using a Synthetic Dataset

For generating the synthetic data, we used the symmetric template mesh constructed using the brain third ventricle binary masks of the aging sample

*Corresponding author

Preprint submitted to Computer Methods and Programs in Biomedicine October 14, 2015

as described in the paper. We, firstly, transformed the template mesh via an anisotropic scale transformation (scale factor: x, y, z = 0.5, 0.9, 0.8) to generate an initial mesh with average width of 2 mm. Afterwards, we added the artificial deformation to the initial mesh. The volume of the initial mesh (549.17 mm^3) is 36% of the template mesh (1525.47 mm^3).

A) Robustness against asymmetric deformations

To validate the robustness of our 3D shape modeling method against asymmetric deformations, we generated a first set of synthetic data by introducing asymmetric deformations to the initial mesh, displacing each vertex of the left halves only to the left. The displacement of the vertices was in a range of 1 mm to 5 mm. Then, we converted the meshes into binary masks with a small voxel size ($0.1 \times 0.1 \times 0.1$ mm^3), thus minimizing the shape representation error produced by the voxelization process. From this overall process we obtained six synthetic binary masks and five deformed meshes, to which we fitted the template mesh via our non-rigid modeling method.

To assess the accuracy of the shape correspondence we manually set five landmarks on the left and right lateral walls of the initial mesh as Figure 1 (a) shows. Then, we compared the landmark positions obtained by our modeling method with those determined by the artificial vertex displacement. We also measured the vertex-wise deformity between the template meshes fitted into the binary mask of the initial mesh and other binary masks.

In addition, on this synthetic dataset, we evaluated the accuracy of the target shape reconstruction using three well-known metrics: 1) the volumetric

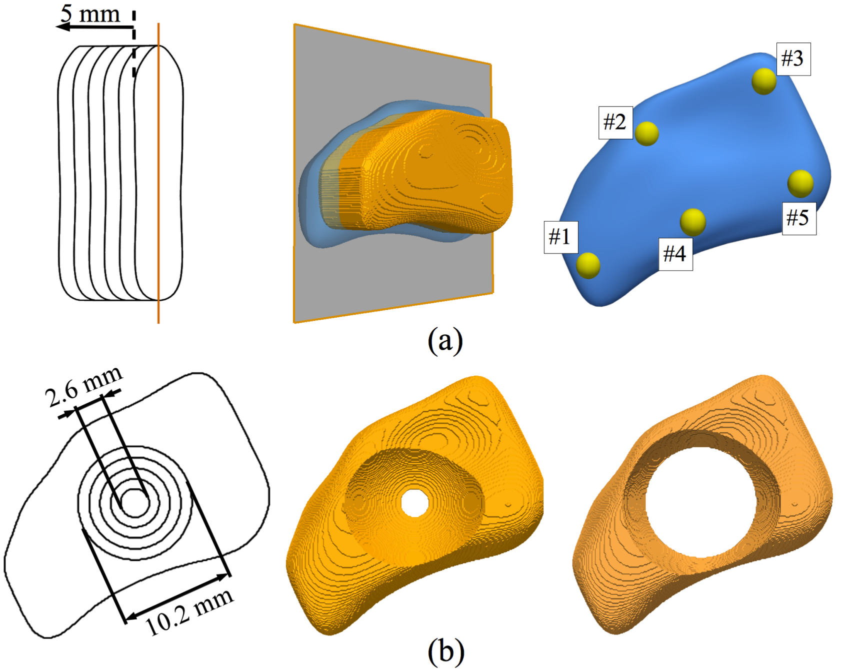

Figure 1: Synthetic data for the robustness validation of the shape modeling method for the third ventricle. The most left figures are the schemes for the synthetic data generation, and the right figures show the generated data. (a) Asymmetric deformation of the left lateral wall of the third ventricle. Yellow circles are the landmarks used for validation. (b) topological variations of the third ventricle in a radius range of 1.3 mm to 5.1 mm. Blue surface is the symmetric template mesh.

similarity index (i.e. Dice coefficient, Dc), 2) the symmetric mean distance (Md), and 3) the symmetric Hausdorff distance (Hd). The Dc quantifies the volume overlap between two volumetric data A and B by:

$$Dc = 2 \cdot \frac{n\left(A \cap B\right)}{n\left(A\right) + n\left(B\right)}$$

where the operator $n\left(\cdot\right)$ returns the volume of the input data and the operator $\cap$ returns the spatially common volume between A and B. The Dc ranges between 0 and 1 representing disjoint and complete overlap respectively. The Md measures the average distance between the voxel meshes of the synthetic binary masks and the fitted template meshes, and the Hd measures the maximum distance between them.

B) Robustness against topological variations

To validate the robustness of our 3D shape modeling method against topological variations, we generated a second set of synthetic data. This second set of synthetic data, was formed by synthetic binary masks constructed simulating various sizes of interthalamic adhesion (IA) passing through the initial mesh. It was achieved by erasing the voxels inside two spheres, initially one at each side of the mesh, which contacted each other at the midplane. The radius of the spheres was 7 mm. By changing the distance between the spheres, we generated synthetic third ventricles with various radii of holes in a range of 1.3 mm to 5.1 mm at the midplane (Figure 1 (b)). Then, we visually compared the modeling results, obtained with and without the midplane-based constraint, and the area of the sampled points, where the left and right surfaces contact each other at the midplane, with the area of

the synthetic holes at the midplane. The area of the sample points of zero width was computed by multiplying the number of sample points to the unit area (0.25 mm^2) of the sampled midplane.

Experiment Results

A) Robustness against asymmetric deformations

Our method generated surface models of high shape similarity (Dc: 0.975 $\pm$ 0.004, Md: 0.068 $\pm$ 0.010 mm and Hd: 0.514 $\pm$ 0.215 mm). Figure 2 (a) shows the positional differences between the corresponding landmarks in the synthetic surfaces and the deformed template meshes. The average of the positional differences was 0.311 $\pm$ 0.123 mm being the minimum 0.092 mm at landmark #1 and the maximum 0.623 mm also at landmark #1. The maximum error at landmark #1 was due to the existence of a sharp corner on the third ventricle's synthetic data, which was generated by the artificial vertex translation, contrary to the smooth curved shape in the template mesh. Nevertheless the positional errors of the landmarks by our method were less than half the voxel size of the T1-weighted MRI of the dataset ($1.0 \times 1.3 \times 1.0 mm^3$). Figure 2 (b) shows the vertex-wise deformity, measured at the landmarks on the left and right lateral walls. The measured deformity values were consistent with the artificial deformation of the third ventricle with only a very small margin of errors, 0.027 $\pm$ 0.024 mm for the left landmarks and 0.043 $\pm$ 0.058 mm for the right landmarks on average. The maximum error in the vertex-wise deformity measurement was 0.292 mm at the landmark #1 on the synthetic data with asymmetric deformation of 5.0 mm.

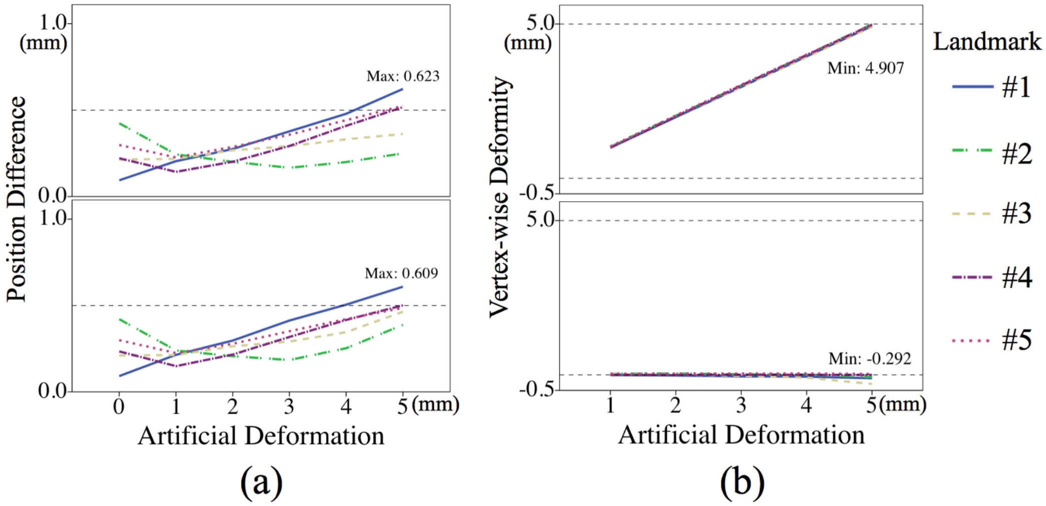

Figure 2: Evaluation results with the synthetic data including the asymmetric deformation of the left lateral wall of the third ventricle. (a) Differences between the landmark positions determined by the synthetic deformation (gold standard) and by the non-rigid template deformation. (b) Vertex-wise deformity between the template meshes fitted into the binary mask of the initial mesh and other binary masks.

Table 1: Comparison between the area of the sample points of zero width and the size of the synthetic IA.

Hole Radius (mm)	1.3	2.2	3.1	4.1	5.1
Ideal IA Area (mm^2)	5.309	15.205	30.191	52.810	81.713
Area of zero width (mm^2)	5.250	15.000	30.750	52.250	79.750
Difference (mm^2)	0.059	0.205	-0.559	0.560	1.963

"Ideal IA area" is the circle size of the hole radius of the synthetic IA.

B) Robustness against topological variations

Figure 3 shows the third ventricle meshes, fitted to the binary masks with synthetic IAs of different sizes. Without the midplane-based constraints (Figure 3 (a)), the template mesh had self-intersection and arbitrary gaps between the left and right lateral walls after the non-rigid deformation. On the contrary, with the midplane-based constraints (Figure 3 (b)), the vertices on the left and right lateral walls of the template mesh were attracted into the midplane of the third ventricle where the IA exists, and they did not cross the midplane. This behavior of the midplane-based constraints made the third ventricle mesh to have zero width where the IA passes through accurately. The area of the sample points of zero width was consistent with the size of the synthetic IA at the midplane with small differences in a range of 0.059 to 1.963 mm^2 (Table 1). The errors were caused by the shape differences between the smooth surface of the deformed meshes and the synthetic data at the edge of the IAs (Figure 3 (c)).

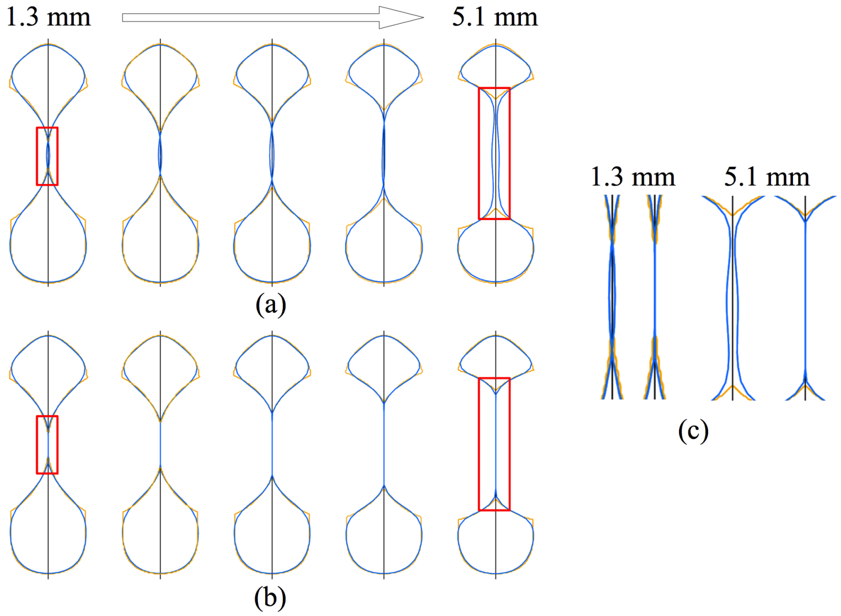

Figure 3: Comparison between the deformed models without (a) and with (b) the midplane-based constraints. Figures show the axial cross sections of the synthetic data (orange) and the deformed meshes (blue). (c) shows the magnified image of the framed area (red).

Conclusion

In this supplementary material, we describe experiments designed to validate our brain third ventricle modeling method against extreme asymmetric shape changes and topological variations using synthetic data. In these experiments, our method showed good enough performance in tracing the asymmetric and complex patterns of the shape changes that can possibly be found in the third ventricle. The evaluation of the feasibility of our method to produce useful and clinically sensible results appears described in details in the main paper.
